# Identifying Influences in Patient Decision-making Processes in Online Health Communities: Data Science Approach

**DOI:** 10.2196/30634

**Published:** 2022-08-31

**Authors:** Mingda Li, Jinhe Shi, Yi Chen

**Affiliations:** 1 Ying Wu College of Computing New Jersey Institute of Technology Newark, NJ United States; 2 Martin Tuchman School of Management New Jersey Institute of Technology Newark, NJ United States

**Keywords:** influence relationship, decision-making threads, online health communities, patient engagement, deep learning, text relevance measurement

## Abstract

**Background:**

In recent years, an increasing number of users have joined online health communities (OHCs) to obtain information and seek support. Patients often look for information and suggestions to support their health care decision-making. It is important to understand patient decision-making processes and identify the influences that patients receive from OHCs.

**Objective:**

We aimed to identify the posts in discussion threads that have influence on users who seek help in their decision-making.

**Methods:**

We proposed a definition of influence relationship of posts in discussion threads. We then developed a framework and a deep learning model for identifying influence relationships. We leveraged the state-of-the-art text relevance measurement methods to generate sparse feature vectors to present text relevance. We modeled the probability of question and action presence in a post as dense features. We then used deep learning techniques to combine the sparse and dense features to learn the influence relationships.

**Results:**

We evaluated the proposed techniques on discussion threads from a popular cancer survivor OHC. The empirical evaluation demonstrated the effectiveness of our approach.

**Conclusions:**

It is feasible to identify influence relationships in OHCs. Using the proposed techniques, a significant number of discussions on an OHC were identified to have had influence. Such discussions are more likely to affect user decision-making processes and engage users’ participation in OHCs. Studies on those discussions can help improve information quality, user engagement, and user experience.

## Introduction

### Background

In recent years, online health communities (OHCs) such as the Cancer Survivors Network (CSN), MedHelp, DoctorLounge, WebMD, and Health-boards message boards have become one of the most important resources that patients leverage [1]. An OHC is defined as an asynchronous web-based message board system for patients that contains multiple message boards, each of which typically focuses on 1 disease. OHCs provide a web-based channel that enables information exchange, facilitates communication, and provides support to patients and caregivers [2-4]. They are especially valuable for patients with chronic diseases to learn about their conditions and seek social support [5,6].

Empowering and supporting patients to make informed health care decisions is a key component of patient-centered health care and is a social, economic, and technical necessity [7,8]. A lot of patients seek information and advice on OHCs. Existing work has found that nearly half of the threads in a breast cancer forum [9] are related to patient decision-making [1]. Studies have also shown that patients are often influenced by web-based sources and social media in their health care decision-making [10,11].

### Objectives

The goal of this study was to identify the influence relationship of posts in discussion threads related to health care decision-making. Specifically, we defined the influence relationships and identified post replies that influenced the initial author, who had questions posted on OHCs.

The outcomes of this study are important for health care professionals to help patients make informed decisions for several reasons. First, analyzing the writing style and pattern of posts that have influence may help explain why they have influence and provide insights to health care professionals on effective communication with patients. Second, if the information provided by posts that have an influence is not accurate, it will mislead patients. It is important to check the information quality in such posts to improve the quality of influence. Furthermore, a patient who has questions but does not receive any replies that have an influence may need further help.

### Literature Review

There is a lot of research conducted on OHC analysis, although with limited study on identifying influence relationships of posts. Several studies have been conducted on analyzing the reciprocal patterns between users’ replies in discussion forums [12-14]. There is also work on analyzing the patterns between post views and post replies [15]. Many studies have been conducted on identifying influential users in a community [16-20]. In those applications, a post, blog, or tweet typically expresses an opinion of the author, and the replies are considered as an indication of being influenced by the opinion of the original post. That is, the reply relationship is considered as an influence relationship. The focus is on judging the influential power of an author based on activeness of post writing [21] and social network features [17,18] such as PageRank-like algorithms or clustering algorithms.

Finding influence relationships among posts in discussion forums is different from finding influential users and requires different techniques. In an OHC, the initial author of a thread typically expresses a question, not an opinion. The influence happens when a reply to the question affects the initial author. There are only 2 existing studies that consider the influence of the replier on the initial author [21,22]. This influence is identified when the sentiment of the initial author is changed to be similar to that of the replier. However, this definition may not be accurate.

**Figure 1:**
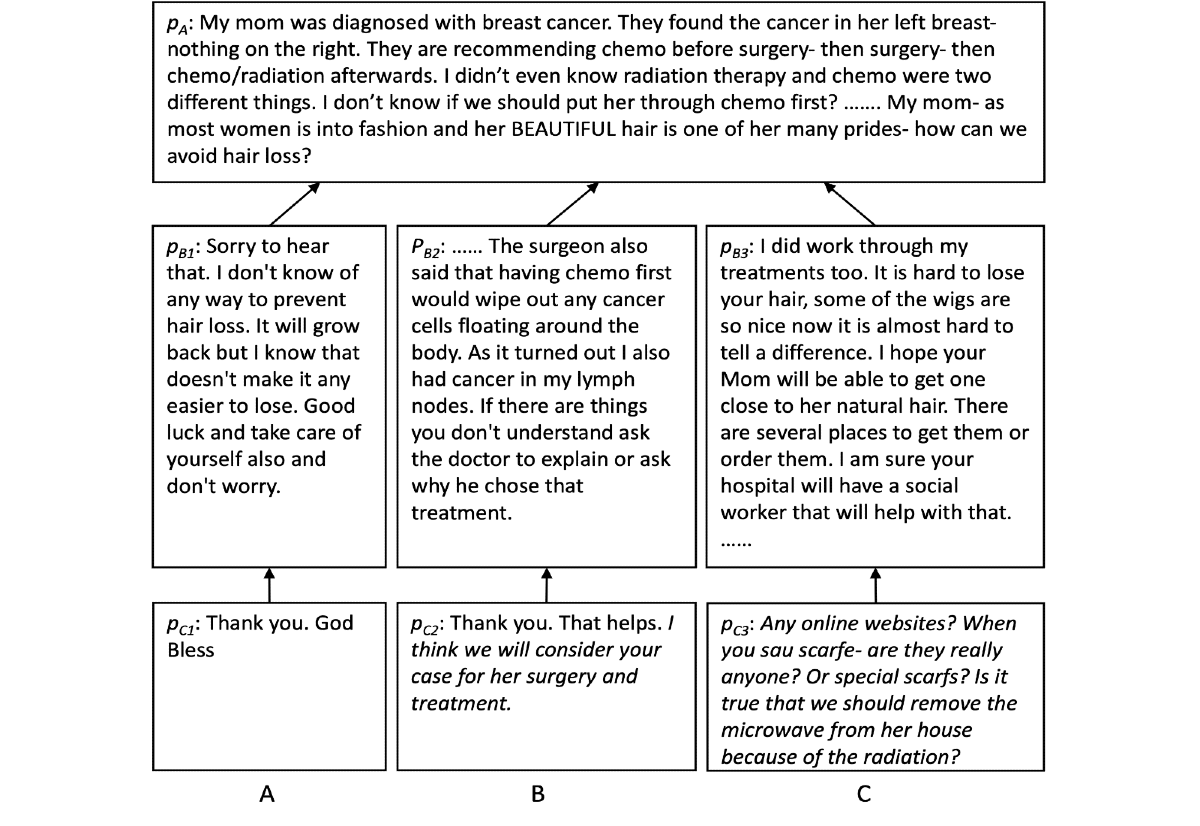
Example of a discussion thread.

Let us look at an example of a discussion thread related to patient decision-making, shown in Figure 1. An OHC user initialized a thread asking for advice on whether to have chemotherapy before surgery for her mother’s treatment plan in post *p_A_*.

In Figure 1A, a user replied by comforting her in post *p_B1_*. The reply was not informative. Even though the initial author expressed gratefulness to the author of post *p_B1_*, with sentiment changing to be positive in post *p_C1_*, she was not influenced by post *p_B1_*. Indeed, studies show that 75% to 85% of CSN forum participants change their sentiment in a positive direction through web-based interactions with other community members [23]. A change in sentiment is not necessarily an indicator of being influenced.

In contrast, in Figure 1B, a user shared her experience in a similar situation suggesting to have chemotherapy before a surgery in post *p_B2_*. The initial author expressed her gratitude and indicated that she would consider this suggestion in determining her mother’s treatment plan (the sentences in italics) in *p_C2_*, showing her being influenced.

### Contribution

Instead of considering sentiment changes, we propose using questions or future actions on relevant replies as an indicator of being influenced, as illustrated in the aforementioned example. There are 2 major challenges in identifying influence relationships. First, we need to define influence relationships of posts. We examined the semantics of post content to define influence relationships. Unlike influential users, who are defined by network features in the existing work [16-20], text content is the key to determine whether posts have influence. Second, it is hard to identify influence relationships. Unlike typical text classification problems, influence relationships involve multiple posts with reply relationships rather than a single paragraph of text. In addition, influence is an abstract concept. It is challenging to extract relevant features to capture the influence patterns considering both content and the reply relationship.

This study makes novel contributions to identifying influence relationships in discussion threads in OHCs related to patient decision-making. Specifically, (1) we defined the influence relationship between the posts based on the semantics of the post content, (2) an extensible deep learning model that extracts and combines both sparse and dense features was proposed to identify the influence relationships in OHC decision-making threads, and (3) the proposed model achieved good performance in identifying influence relationships in empirical evaluation.

## Methods

In this section, we first model the OHC data and define the influence relationship in discussion threads. We then propose a deep learning–based model to identify the influence relationships.

### Problem Definition

#### Definition of Discussion Threads

**Figure 2:**
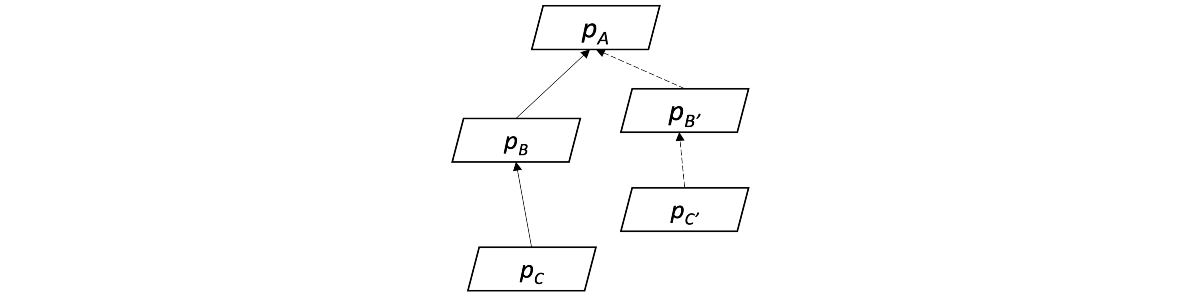
Data structure of an online health community.

Figure 2 presents an overview of the OHC data structure. We modeled an OHC as a set of discussion threads *T* = {*t_1_, t_2_,..., t_n_*}. Each thread *t_i_* is composed of a set of posts and a function *R* that represents the reply relationship. For example, Figure 2 illustrates a thread that contains a set of 5 posts {*p_A_, p_B_, p_C_, p_B’_, p_C’_*}. One of the reply relationships, *R*(*p_B_*) = *p_A_*, represents that post *p_B_* replies to post *p_A_*. Each post *p_i_* consists of a sequence of sentences *p_i_* = {*s_1_*, *s_2_*,..., *s_l_*}. Each post has an author. We denoted the author relationship using a function *U*. *U*(*p_i_*) represents the author of post *p_i_*. Note that a post only has a single author; however, an author may write ≥0 posts in a thread. We used *p_A_* to present the first post of a thread and named it the *initial post*. The author of the *initial post*, *U*(*p_A_*), is referred to as the *initial author* of the thread.

Existing work [1] has studied the thread discussions in OHCs and identified that a subset of threads is related to *patient decision-making*. Such a thread is characterized by questions in the initial post and replies with suggestions of options. Techniques have been developed to identify decision-making threads in OHCs.

In this paper, we study how to identify the cases where the initial author of a decision-making thread is influenced by a reply post. Note that our study is general to any thread discussions related to decision-making. The definition and identification of decision-making threads can be handled using the approach developed in existing work [1] or other approaches. In the rest of this paper, we use *threads* to refer to decision-making threads for simplicity. The defined influence relationship may not be applicable to discussion threads that are not related to decision-making, such as discussion threads for casual communication or experience-sharing threads providing social support.

#### Definition of Relationships

##### Overview

Before introducing the definition of *influence relationships*, we first introduce relationships. A relationship is defined on a triple of posts in a thread with reply relationships: an initial post, a reply to the initial post, and the initial author’s subsequent reply.

##### Definition 1 (Relationship)

We define the relationship among three posts *p_A_*, *p_B_*, and *p_C_*, in a thread as *r_i_* = (*p_A_*, *p_B_*, and *p_C_*), where post *p_A_* is the initial post of the thread, post *p_B_* replies to *p_A_*, post *p_C_* replies to *p_B_*, and the authors of *p_A_* and *p_C_* are the same person. That is, *R*(*p_B_*) = *p_A_, R*(*p_C_*) = *p_B_,* and *U*(*p_A_*) = *U*(*p_C_*).

We used *r_i_* = (*p_A_, p_B_, p_C_*) to denote the relationship among *p_A_, p_B_,* and *p_C_*. Note that there are many such relationships in a thread, and we considered all such triples. For instance, Figure 2 shows a thread with 2 relationships, *r_1_* = (*p_A_, p_B_, p_C_*) and *r_2_* = (*p_A_, p_B’_, p_C’_*).

Also, note that existing work on identifying influential users [16-20] does not consider the relationships among post triples but only considers the reply relationship between 2 posts.

#### Definition of Influence Relationships

##### Intuition

Now, we discuss how to define *influence relationships* on relationship (*p_A_, p_B_, p_C_*), where post *p_B_* has an influence on the initial author *U*(*p_A_*).

First, intuitively, if post *p_B_* influences the initial author *U*(*p_A_*), then the content of these 3 posts must be relevant.

Second, we referred to the definition of *influence* in Merriam-Webster [24]—“to affect or alter by indirect or intangible means”—and the reaction of *being influenced* is to *sway* rather than being convinced. If the initial author considers the suggestion given in post *p_B_*, even if she eventually does not take the suggestion, she is considered to have been influenced by post *p_B_.* On the basis of this definition, we observed 2 indications that the initial author, *U*(*p_A_*), was influenced by *p_B_*.

An observation of being influenced is that the initial author may ask questions in *p_C_* based on the suggestions in *p_B_*. Curiosity is a motivator for learning and influential in decision-making [25]. An existing study [26] used a statistically large sample of learning forum posts to investigate whether student participation in the forum could be influenced. They observed that students who were influenced by others’ interesting answers were more likely to ask follow-up questions. This indicates that asking further questions is a sign of being influenced. The same pattern also exists in OHCs. Let us look at the example in Figure 1C. The initial author expressed concerns about hair loss in *p_A_*. Another user replied in post *p_B3_* suggesting the use of wigs. The initial author then replied in post *p_C3_* with questions (the sentences in italics) for more details about the suggestion given in post *p_B3_*. These questions indicate that the initial author was thinking about the suggestion given in post *p_B_*; that is, being influenced.

The second indication that the initial author was influenced by a post *p_B_* is that she expressed her intention to take action in post *p_C_*. Adjei et al [27] found that member-to-member communication in web-based brand communities greatly influenced the members’ future purchase behavior. Similarly, the communication through discussion threads in OHCs may also affect the initial author’s future actions. Let us look at the example in Figure 1B again. For the treatment question asked in *p_A_*, a forum user shared her experience and discussed the treatment in post *p_B2_*. The initial author then replied with a planned action (the sentence in italics) in *p_C2_*. The intention of future action based on the communications in the thread is an indicator of the influence relationship.

On the basis of these observations, we define influence relationships in decision-making threads in the following section.

##### Definition 2 (Influence Relationship)

A relationship *r_i_* = (*p_A_, p_B_, p_C_*) is considered as an influence relationship—that is, *U*(*p_A_*) is influenced by *p_B_*—if and only if the following conditions are met: (1) the content of *p_B_* is relevant to post *p_A_*, (2) the content of *p_C_* is relevant to post *p_B_*, and (3) *p_C_* contains questions or indicates future actions.

To identify influence relationships, we modeled it as a classification task. Given a set of relationships *R* = {*r_1_, r_2_,..., r_n_*}, for each relationship *r_i_*, we predicted its label to be either 1 or −1, where label 1 indicated that *r_i_* was an influence relationship and label –1 indicated that *r_i_* was not an influence relationship. The goal was to learn a model from the labels of known relationships and predict the labels for unlabeled relationships.

### Model Design

#### Overview

**Figure 3:**
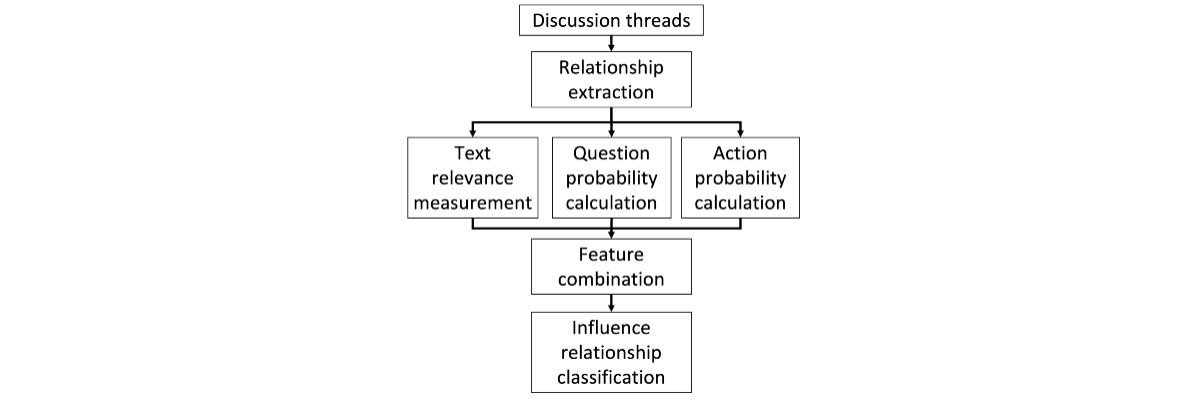
Workflow of influence relationship identification.

In this section, we present the method to identify the influence relationships in decision-making threads in OHCs. Figure 3 presents the framework of the proposed method.

Given a set of discussion threads as the input, we first extracted the triple relationships using the relationship extraction module. Text relevance features, question probability features, and action probability features were then calculated using the text relevance measurement module, the question probability calculation module, and the action probability calculation module, respectively. Finally, all the features were combined using a deep learning model in the feature combination module to generate the probability of a relationship being an influence relationship.

#### Relationship Extraction Module

In this section, we introduce the relationship extraction module, which extracted all relationships defined in definition 1.

In the first step of relationship extraction, we built the reply tree structure based on the indented format in html files. For each adjacent post pair, the post that was posted earlier was treated as the parent of the latter post. The ancestor-descent distance between a post and the initial post was represented by the number of tab characters. The reply structure of a thread is illustrated in Figure 2. Each post is a node in the thread tree, and each edge represents a reply relationship. The root of the thread tree is the initial post (ie, *p_A_*) in definition 1.

Existing work observes that, in some forums, the reply structure in a discussion thread may not be fully available and proposes techniques to construct full reply structures [28]. The OHCs used in our experiments had a full reply structure. Existing techniques can be leveraged if needed for other forums.

We then navigated the thread tree to extract all relationship triples, as defined in definition 1. Each triple started with the initial post followed by a reply to the initial post written by another author and then a subsequent reply by the initial author, all of which were on the same path in the thread tree. For example, *r_1_* = (*p_A_, p_B_, p_C_*) and *r_2_* = (*p_A_, p_B’_, p_C’_*) are 2 relationships in the thread tree in Figure 2.

#### Text Relevance Measurement Module

The text relevance measurement module measures the content relevance, or text semantic similarity, of 2 posts using a relevance score between 0 and 1.

There are mainly 2 types of deep learning–based methods in the literature that measure text relevance. The first type of method extracts content feature vectors of 2 input texts and then combines them to make a prediction, such as the Deep Structured Semantic Models (DSSM) [29], the Convolutional DSSM [30], and Architecture-I (ARC-I) [31]. The intuition of this method is to highlight the important information of the original texts so that irrelevant content can be removed before the feature combination phase. However, the drawback of this type of method is that it runs the risk of losing detail [32].

The second type generates the word-level relevance first and then uses neural networks to learn the hierarchical interaction patterns for content-level relevance, such as DeepMatch [33], Architecture-II (ARC-II) [31], and MatchPyramid [34]. The motivation is that making a good relevance judgment requires considering the interactions in the text relevance measurement process, starting from the interactions between words to patterns in phrases and those in whole sentences [34]. However, the training process for the second type is much more expensive than for the first one.

We evaluated both approaches to measure text relevance in experiments. We chose 2 state-of-the-art representative methods for the text relevance measurement module in the evaluation. For the first type, we chose ARC-I [31], which uses a multilayer perceptron to combine relevance feature vectors. It shows better performance than the DSSM [29] and Convolutional DSSM [30], both of which use cosine similarity [34]. We chose MatchPyramid [34] to represent the second type of method as it exhibits better performance than the other 2 methods (DeepMatch [33] and ARC-II [31]) in experiments on multiple data sets [34].

We further proposed the adaptation of Bidirectional Encoder Representations from Transformers (BERT) [35] as the embedding layer in the ARC-I and MatchPyramid models. BERT is a state-of-the-art embedding method for word representation in many natural language understanding tasks, trained on BookCorpus and English Wikipedia. We considered both BERT (trained on Wikipedia) and word2vec (trained on the training data set) as the embedding methods for both ARC-I and MatchPyramid. Different variations of the text relevance measurement module are evaluated in the *Text Relevance Evaluation* section.

#### Question Probability Calculation Module

We now discuss how to calculate the probability of a post containing a question using the question probability calculation module.

There are 2 types of methods to identify question sentences in forums: a rule-based approach and a learning-based approach. In a rule-based approach, question marks and 5W1H words (what, who, when, where, why, and how) are used to identify question sentences [36]. A learning-based approach uses sequential question patterns to train a binary classifier on labeled data [37-40]. Liu and Jansen [37] used the question mark to extract question posts from Sina Weibo. In the studies by Ranganath et al [38,39], frameworks were proposed to identify rhetorical questions by modeling the motivation of the user for posting them. In the study by Ojokoh et al [40], questions from ResearchGate were identified based on the maximum probability value of a naïve Bayes classification with part-of-speech tag features.

Both rule-based and learning-based approaches can achieve excellent performances. A study shows that a rule-based approach can outperform complicated learning-based approaches [36]. Thus, we followed a rule-based method [36] to identify question presence in the posts. In total, 2 types of rules were considered: question marks and 5W1H words. We made adaptations of this approach for OHCs. As a question mark is the most significant sign of a question, we gave a higher confidence score to a sentence with a question mark. We also set some constraints on 5W1H words to simulate the pattern of question sentences. First, 5W1H must appear at the beginning of a sentence. Second, auxiliary words were added to the original words for more specific patterns. For example, we considered *what is*, *what are*, *what does*, and *what do* instead of *what*.

After the question probability of each sentence in a post *p_i_* was calculated, the maximum probability was used as the likelihood of post *p_i_* containing at least one question, denoted as *Q*(*p_i_*).

#### Action Probability Calculation Module

This section presents the action probability calculation module, which generated the probability of action presence in a post.

The indication of a future action can be captured by the presence of verbs and appropriate sentence tense. The Natural Language Toolkit (NLTK) [41] tagger module defines a standard interface for augmenting each token of a text with supplementary information, such as its part of speech or its WordNet synset tag, and provides several different implementations for this interface. We leveraged the NLTK tagger module to assess the likelihood of a post containing future actions by checking the existence of words with a future tense verb tag (eg, *will consider* in Figure 1B) or a modal auxiliaries tag (eg, *can*, *could*, *may*, and *must*). To count on the cases where future tenses may not be identified because of forum users’ typos or informal writing, we set the probability of future action to be 0.5 when the rules failed to identify future actions. Equation 1 shows the calculation formula to generate the action probability of a post *p_i_*.

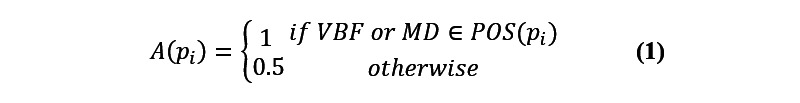



Note that we did not consider negation in the action probability calculation module. For example, in post *p_C_*, the initial author disagrees with the suggestions proposed in *p_B_* and decides to do something different. For those cases, the overall meaning of *p_B_* and *p_C_* would be the opposite and, therefore, would be captured by the relevance vectors generated in the text relevance measurement module. Thus, we did not consider negations in this phase to avoid double counting.

#### Feature Combination Module

##### Overview

**Figure 4:**
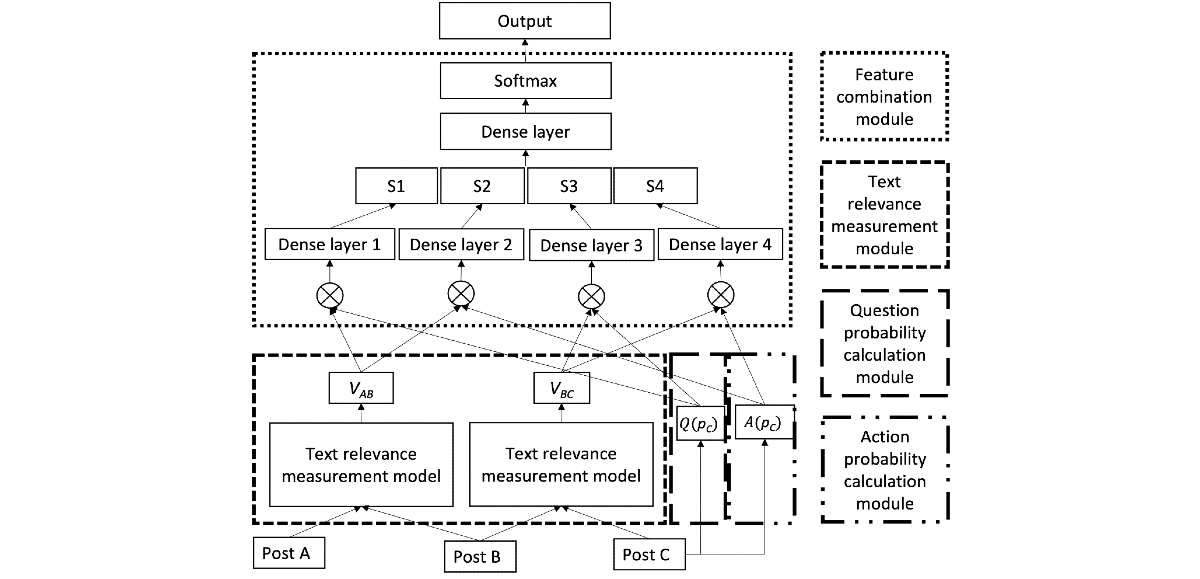
Architecture of the feature combination module.

Referring to Figure 4, the text relevance measurement module calculated *P_AB_*—the relevance score between *p_A_* and *p_B_*—and *P_BC_*—the relevance score between *p_B_* and *p_C_*. The question probability calculation module and action probability calculation module calculated the question probability *Q*(*p_C_*)—or *Q* in short—and action probability *A*(*p_C_*)—or *A* in short—based on the text of *p_C_*.

We now discuss the feature combination module that measures the influence score based on these features. We discuss 2 alternative methods: *a baseline approach* and *a deep learning model*.

##### Baseline Approach

Recall that, according to definition 2, the presence of an influence relationship requires the relevance between post *p_A_* and post *p_B_*, the relevance between post *p_B_* and post *p_C_*, and the presence of a question or action in post *p_C_*. We started with an intuitive method to detect influence relationships based on the definition using Equation 2.

*P_baseline_* = *P_AB_* × *P_BC_* × max [*Q*(*p_C_*), *A*(*p_C_*)] **(2)**

We set the thresholds to 0.5, 0.5, and 0.9 for each component.

##### Deep Learning Approach

We further proposed a deep learning model that combines the text relevance, the likelihood of question presence, and the likelihood of future action presence to identify influence relationships. The architecture of this model is shown in Figure 4.

Compared with the *baseline* approach, there are 3 major benefits of using a deep learning model. First, it is labor-intensive, time-consuming, and difficult to determine appropriate thresholds for cutting off the probabilities using a rule-based approach such as the *baseline* approach. A threshold that works well for one data set may not be optimal for another. Both a rule-based approach and a deep learning model require different thresholds for different data sets. A rule-based approach requires manual parameter tuning for each data set. In contrast, a deep learning approach learns thresholds from the ground truth and, thus, can easily adapt to a new data set with minimal human intervention [42]. Second, the question and action features may have different interactions with the relevance features. We observed that questions are often relevant, but actions are not necessarily. People typically express appreciation in post *p_C_* or sometimes even mention actions totally irrelevant to post *p_B_*, such as the plan to travel or shop. Being relevant is more important to consider in the presence of actions compared with in the presence of questions. However, in the *baseline* approach, the question and action features are merged before being combined with the relevance features, resulting in the loss of important information. Furthermore, we used relevance vectors as inputs to the deep learning model to calculate the influence score. Compared with the *baseline* approach, which uses the relevance scores as input to measure the influence score, relevance vectors provide much richer information. This can be especially helpful when there are several topics involved in the discussion. The relevance information is also leveraged during the phase of combining the relevance features with the question or action features.

Let *V_AB_* denote the relevance vector between *p_A_* and *p_B_* and *V_BC_* denote the relevance vector between *p_B_* and *p_C_*. We generated *V_AB_,V_BC_* from *p_A_*, *p_B_*, and *p_C_* and calculated *Q* and *A* from *p_C_*.

These features were then connected. The question or future action in *p_C_* must be related to the content of *p_A_* and *p_B_*. Thus, we combined *V_AB_* and *V_BC_* with *Q* and *A* using one of the following two operators: (1) *cat* (concatenating each relevance vector with question or action probability) and (2) *dot* (multiplying each relevance vector with question or action probability).

There are 2 major differences between these 2 operators for connecting the features: *cat* and *dot*. First, *dot* makes sure that *Q* and *A* affect each dimension in the relevance vectors, whereas *cat* cannot guarantee this as some neurons or nodes are dropped out. Some interactions between questions or actions and text relevance may be ignored by the *cat* operator. Second, the training process of the *cat* is more expensive than that of the *dot* because, for each dense layer 1 to 4, there is an additional dimension for the *cat* compared with for the *dot*.

In Figure 4, we use ⊗ to present the combination operator, which can be either *cat* or *dot*. The combination step produces 4 feature vectors: *V_AB_* ⊗ *Q*, *V_AB_* ⊗ *A*, *V_BC_* ⊗ *Q*, and *V_BC_* ⊗ *A*. To extract the key information from these combined feature vectors, 4 dense (fully connected) layers were used to populate the summarized feature vectors (*S_1_, S_2_, S_3_, S_4_*). The concatenation of these 4 summarized feature vectors was passed through 2 dense layers. The first one was used to further combine the summarized feature vectors. The second one aimed to generate the probability distribution over the labels. To avoid gradient vanishing and exploding [43], we chose the *Relu* function as the activation function for all the dense layers except the output layer, which uses the *softmax* function to populate the probabilities.

We trained the model using the binary cross-entropy loss function defined in Equation 3, which minimizes the distance between the probability distributions of the ground truth and those of the predicted score.

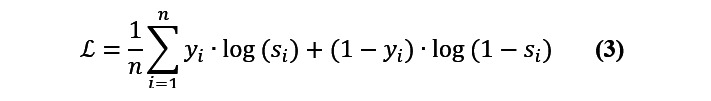



Where *y_i_* is the ground truth label of the *i*th training sample and *s_i_* is the score predicted by the model. The Adam optimizer [43] was leveraged for optimization because of its advantage of processing sparse features and obtaining faster convergence compared with the normal stochastic gradient descent with momentum.

### Ethics Approval

All materials were obtained from anonymous open-source data. Thus, ethics approval was not required.

## Results

### Experiment Setting and Evaluation Metrics

We implemented a prototype system for influence relationship identification on discussion threads. The prototype system and data sets used in the evaluation are publicly available at GitHub [44].

For empirical evaluation, we collected 25,208 threads that were publicly available in the CSN breast cancer forum [9]. The webpages were collected and processed by a web crawler we developed leveraging the Spider Crawler library [45]. There were 321,000 posts with 1.9 million sentences in total. We applied the classifier proposed by Li et al [1] on all 25,208 threads to identify the ones that were related to patient decision-making and obtained 11,815 (46.87%) such threads. Note that other models for classifying decision-making threads can also be plugged in.

We then extracted relationships from the decision-making threads using the relationship extraction module and obtained 9053 relationships. We randomly picked 853 (9.42%) of them to label. A total of 4 PhD students worked on the manual labeling. All the relationship triples and post pairs were first independently labeled. In case of disagreement, a consensus was reached after discussion. A total of 261 relationships were labeled as influence relationships. Recall that, per definition 1, each relationship is presented as a triple (*p_A_, p_B_, p_C_*). We also labeled whether posts *p_A_* and *p_B_* were relevant (ie, *P_AB_*) and whether posts *p_B_* and *p_C_* were relevant (ie, *P_BC_*). We observed some reply posts with content expressing only comfort or wishes. Although they express care about the initial author’s conditions and seem relevant, they are generic. After discussion, we reached an agreement that, when the initial post and reply post shared similar medical terms (such as *chemotherapy* and *chemo*), we would label them as relevant. All 1706 post pairs (*p_A_, p_B_*) and (*p_B_, p_C_*) of the 853 relationships were labeled. Of the 1706 pairs, 1210 (70.93%) were relevant pairs, and the remaining 496 (29.07%) were irrelevant. We split the set of relationships into a training set (90%) and a testing set (10%). The post pairs in the aforementioned training and test sets were used for text relevance training and testing, respectively.

The metrics used for evaluation included precision, recall, F_1_ score, accuracy, area under the receiver operating characteristic curve (ROC AUC), and area under the precision-recall curve (PR AUC). They evaluated the effectiveness of a system using different aspects: (1) *precision*, also known as positive predictive value, is the fraction of relevant instances among the retrieved instances; (2) *recall*, also known as sensitivity, is the fraction of relevant instances that are retrieved among all relevant instances; (3) *F_1_ score* measures a model’s performance by calculating the harmonic mean of the precision and recall, as shown in the following equation: 
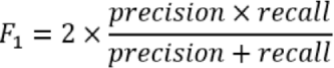
 **(4)**; (4) *accuracy* is a common evaluation metric for binary classification problems and is defined as the fraction of corrected predictions among the total number of predictions; (5) *ROC AUC* is a common evaluation metric for binary classification problems and is created by plotting the true positive rate against the false positive rate at various threshold settings; and (6) *PR AUC* is commonly used to evaluate the performance of a model on data sets with imbalanced labels.

### Text Relevance Evaluation

Table 1 presents the classification results of the text relevance measurement module. In total, 2 observations were made. The first observation was that the models using BERT achieved high recall but low precision, whereas the models with word-embedding vectors trained on OHC data obtained balanced precision and recall values. There are 2 reasons for these results. First, OHC data are domain-sensitive and can benefit from domain-specific word representation. Second, the BERT transformer tends to link words in adjacent sentences by mistake. In the text relevance measurement module, precision was more important than recall as the accuracy of influence relationship identification depended on the precision of relevance classification. Thus, we used the word vectors trained on OHC data instead of BERT in the following experiments.

**Table 1:** Text relevance measurement module results.

	Precision	Recall	F_1_	Accuracy	ROC AUC^a^	PR AUC^b^
MatchPyramid with BERT^c^ (trained on Wikipedia)	0.578	*0.992* ^d^	0.730	0.512	0.502	0.583
MatchPyramid with word2vec (trained on the training data set)	0.781	0.820^d^	*0.806*	0.692	0.763	0.854
ARC-I^e^ with BERT (trained on Wikipedia)	0.523	0.890^d^	0.659	0.503	0.493	0.554
ARC-I with word2vec (trained on the training data set)	*0.832*	0.747^d^	0.785	*0.784*	*0.848*	*0.903*

^a^ROC AUC: area under the receiver operating characteristic curve.

^b^PR AUC: area under the precision-recall curve.

^c^BERT: Bidirectional Encoder Representations from Transformers.

^d^The *P* value is statistically significant at *P*=.05.

^e^ARC-I: Architecture-I.

The second observation was that, with word vector embedding, ARC-I achieved a better performance than MatchPyramid in most of the evaluation metrics. In the ARC-I model, each input text goes through an embedding layer, a convolution layer, and a max pooling layer, and the extracted feature vectors are then concatenated together as the input to a fully connected layer that calculates the predicted relevance scores. MatchPyramid populates the local word relevance matrix first. Each cell of the matrix presents the dot product of the word-embedding vectors of the words in the text input. The patterns of these interactions are then extracted using a convolutional neural network [46]. Thus, ARC-I focuses on checking relevance based on the meaning of the whole text, whereas MatchPyramid focuses on summarizing the important relevance features based on local word similarity. For OHC data sets, posts were relatively long and often contained noisy information; thus, considering the meaning of the entire post text was more important than focusing on adjacent words. This is why the performance of ARC-I was better than that of MatchPyramid in our evaluation. We also observed that *ARC-I* with word2vec outperformed *MatchPyramid* *with word2vec* in both *ROC AUC* and *PR AUC* but had an inferior *F_1_ score*. Note that F_1_ averages the performance of all the samples by combining the precision and recall, whereas the ROC AUC and PR AUC cumulate the precisions among all samples with different recall thresholds. This indicates that the average performance of *MatchPyramid* *with word2vec* was better, but the overall performance of *ARC-I* *with word2vec* was better.

### Question and Action Probability Evaluation

Now, we present the evaluation of the question probability calculation module and the action probability calculation module. The performance is shown in Table 2. Good performance was achieved for question identification. For future action identification, a high score was achieved on recall but not on precision. The following are a few examples of posts that are classified as containing future actions but actually do not have action intent: *I will tell you though I hated my silicone* or *I would worry about it*. These sentences have verbs in the future tense, but those verbs only convey opinions or feelings rather than taking action on health care. We plan to improve action detection by training action sentence models as future work.

Recall that in the *deep learning* approach, question and action probabilities are considered as input features instead of imposing a strict requirement on their presence. We conducted an analysis on the test data in terms of their presence. All positive cases either had a probability of action presence of 1.0 or had a high probability of question presence, with an average probability of 0.986 (SD 0.033). This indicates that the *deep learning* approach captures definition 2 well, ensuring the high likelihood that either a question or a future action is present.

**Table 2:** Question and action calculation module results.

	Precision	Recall	F_1_	Accuracy	ROC AUC^a^	PR AUC^b^
Question probability calculation module	*1.000*	*1.000* ^c^	*1.000*	*1.000*	*1.000*	*1.000*
Action probability calculation module	0.771	*1.000* ^c^	0.871	0.810	0.733	0.771

^a^ROC AUC: area under the receiver operating characteristic curve.

^b^PR AUC: area under the precision-recall curve.

^c^The *P* value is statistically significant at *P*=.05.

### Influence Relationship Classification Evaluation

Table 3 shows the performance of the *baseline* and *deep learning* approaches with alternative ways to combine text relevance vectors, question features, and action features. Recall that, for the feature combination module, *baseline* combines the text relevance score, the likelihood of question presence, and the likelihood of future action presence to identify influence relationships. *MatchPyramid+cat Q/A* represents the model using *MatchPyramid* to calculate the text relevance score and *cat* as the combination operator ⊗, whereas *MatchPyramid+dot Q/A* uses *dot* as the combination operator ⊗. *ARC-I+cat Q/A* represents the model using *ARC-I* to calculate the relevance score and *cat* as the combination operator ⊗, whereas *ARC-I+dot Q/A* uses *dot* as the combination operator ⊗.

**Table 3:** Influence relationship classification results.

	Precision	Recall	F_1_	Accuracy	ROC AUC^a^	PR AUC^b^
Baseline	0.300	0.231^c^	0.261	0.595	0.495	0.307
MatchPyramid+cat Q/A^d^	0.667	0.154^c^	0.25	0.714	0.560	0.442
MatchPyramid+dot Q/A^e^	0.633	*0.577* ^c^	*0.603*	0.667	0.634	0.481
ARC-I+cat Q/A^f^	0.667	0.154^c^	0.25	0.714	0.637	0.515
ARC-I+dot Q/A^g^	*0.750*	0.462^c^	0.571	*0.786*	*0.724*	*0.631*

^a^ROC AUC: area under the receiver operating characteristic curve.

^b^PR AUC: area under the precision-recall curve.

^c^The *P* value is statistically significant at *P=*.05.

^d^MatchPyramid+cat Q/A: model using *MatchPyramid* to calculate the text relevance score and *cat* as the combination operator ⊗.

^e^MatchPyramid+dot Q/A: model using *MatchPyramid* to calculate the text relevance score and *dot* as the combination operator ⊗.

^f^ARC-I+cat Q/A: model using *Architecture-I* to calculate the relevance score and *cat* as the combination operator ⊗.

^g^ARC-I+dot Q/A: model using *Architecture-I* to calculate the relevance score and *dot* as the combination operator ⊗.

**Figure 5:**
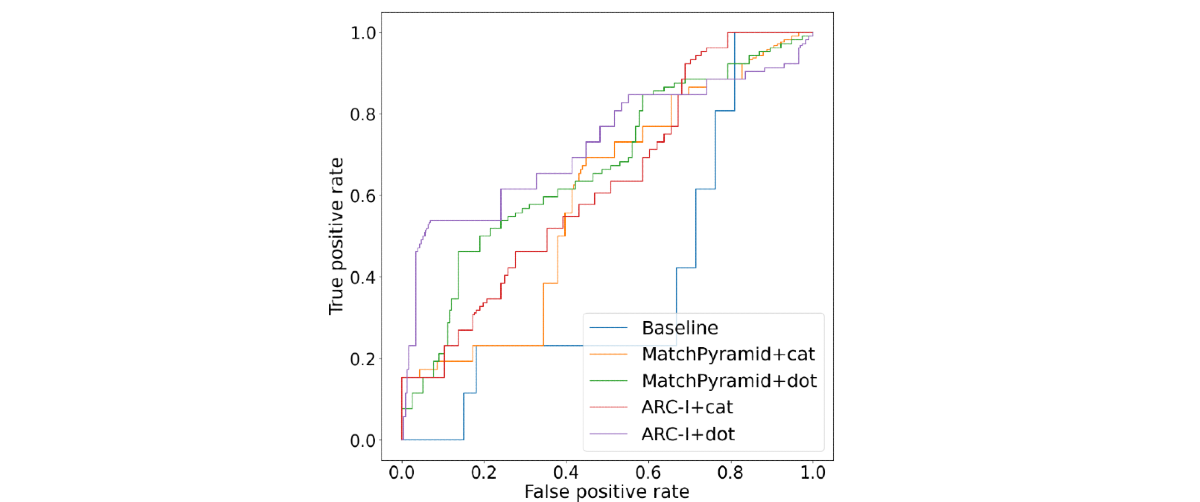
Influence relationship classification.

We also visualized the operating characteristic curves of all methods, as shown in Figure 5. From Table 3 and Figure 5, we have the following observations.

First, all proposed *deep learning* methods, which use relevance features and consider the interaction between relevance and the presence of questions or actions, significantly outperformed the *baseline* approach. This indicates that the relevance feature vectors generated by the text relevance measurement module were effective in capturing relevant content. Combining these feature vectors with the features of question presence and action presence helped capture their interactions and achieved good performance in influence relationship classification. In contrast, the *baseline* approach, which directly follows definition 2, did not perform well. This was due to the inability to capture the interactions between text relevance and question or action presence and the challenge of manually setting an appropriate cutoff threshold for each module.

Second, the models using the *dot* operator performed better than those using the *cat* operator. There are mainly 2 reasons for this. First, question probability and action probability may interact with *V_AB_* and *V_BC_* relevance vectors, which can be captured well by the *dot* operator. Figure 1B shows an example in which the action in *p_C_* is related to the discussion in *p_A_* and *p_B_*. The action in *p_C2_* is related to *chemo*, which is the common content of *p_A_* and *p_B2_*. In this case, the action probability needs to be combined with *V_AB_*. Although, in another case, the action refers to an option mentioned in *p_B_*, the interaction between *p_B_* and *p_C_* is more likely to be the context of the action and, thus, the action probability needs to be combined with *V_BC_*. In contrast, the *cat* operator ignores some interactions between questions (actions) and the context because of the dropout of some neutrals. Therefore, the *cat*-based methods had a much lower recall than the *dot*-based methods. The results show that interactions between action and context are important for influence identification.

Furthermore, the *ARC-I+dot Q/A* had a much better precision, accuracy, ROC AUC, and PR AUC than *MatchPyramid+dot Q/A* but had lower recall and slightly lower F_1_. This is because ARC-I achieved a better performance than MatchPyramid in the text relevance measurement module. *ARC-I+dot Q/A* was stricter than *MatchPyramid+dot Q/A* when fitting the model to the relevance factor. For applications that want to analyze the writing style and patterns of posts that have influence, precision is critical. *ARC-I+dot Q/A* is effective for locating such discussions. In contrast, for applications that want to check the information quality of the posts that have influence to prevent and mitigate the spread of misleading information, *MatchPyramid+dot Q/A* is more suitable because of its higher recall.

### A Case Study

Figure 1 shows an example of 3 relationships, (*p_A_, p_B1_, p_C1_*), (*p_A_, p_B2_, p_C2_*), and (*p_A_, p_B3_, p_C3_*), where (*p_A_* is the initial post of the thread. The scores of these 3 relationships calculated using our system were 0.282, 0.793, and 0.622, respectively. Our system identified (*p_A_, p_B2_, p_C2_*) and (*p_A_, p_B3_, p_C3_*) as each containing an influence relationship, and (*p_A_, p_B1_, p_C1_*) does not. As we can see from the post content, *p_B2_* provides suggestions to the initial author regarding the treatment decision. In post *p_C2_*, the initial author expresses actions to take based on the suggestions in *p_B2_*. In post *p_B3_*, the replier recommends that the author use wigs. The initial author then asks further questions about the wig information. Both relationships indicate that the initial author was influenced. In contrast, *p_B1_* discusses general information and comforts the initial author, and the initial author expresses thanks in *p_C1_*, but there is no indication of being influenced.

## Discussion

### Principal Findings

To the best of our knowledge, this is the first study that defines the influence relationships of discussion posts related to decision-making in OHCs. We proposed a deep learning–based natural language processing prototype to identify influence relationships. We then applied the developed techniques to identify the influence relationships in an OHC, the CSN breast cancer forum. There were 2 major observations.

First, we found that there is a significant amount of influence relationships in the OHC. Of the 9052 relationships in decision-making threads identified by Li et al [1], 3069 (33.9%) were identified as influence relationships. That is, approximately one-third of the communications influence the initial authors on their decision-making. Furthermore, of the 5143 decision-making threads, which have at least one relationship, 2417 (47%) contain at least one influence relationship. Owing to the prevalence, it is important to study posts that have influence.

Second, we also observed that posts that have influence may contribute to engaging users in discussions. The average number of posts in threads containing at least one influence relationship was 15.5, whereas the average number of posts in threads containing no influence relationship was 12.6. Our conjecture is that posts that have an influence likely provide helpful information or good reasoning, which are thought-provoking and help engage users in discussions.

On the basis of these observations, there are several applications that can benefit from the identification and analysis of influence relationships.

First, analyzing the quality of posts that have influence helps improve the quality of the influence. As discussed in the first observation, influence relationships are common. Quality checking of those posts is more critical than that of other posts in terms of improving the effect of influences and mitigating the spread of misleading information.

On the basis of the identification of influence relationships, we can further identify influential users in OHCs. We can use existing techniques that analyze the network features to identify influential users [16-20], where this work calculates the edge weights (ie, the influence of a post). Identifying and checking influential users contributes to high-quality information dissemination.

Second, based on the second observation, analyzing the writing style of posts that have influence provides insights to health care professionals about effective communication for patient engagement.

Furthermore, identifying influence relationships contributes to effective information recommendations for addressing the information overload problem. When a user searches for information in OHCs, it is important to rank discussion threads and posts and recommend to users the most relevant and helpful discussions. On the basis of the analysis of influence relationships and the second observation, discussions that contain influence relationships are more likely to provide helpful information and encourage patient engagement. Thus, the presence of influence relationships is a positive factor in ranking.

### Limitations

Our results are not without limitations. First, our definition of relationship was based on 3 posts, including the initial post in the thread. Therefore, we only identified the posts that had an influence on the initial author. However, any 3 posts that have a sequential reply relationship with the first and third posts from the same author can represent a relationship. We conjecture that the proposed techniques can be used to identify influence relationships among the generalized relationships and plan to study that problem in the future. Second, in this study, we considered text relevance between the posts in the relationship. Sometimes, even though 2 posts, *p_B_* and *p_C_*, are relevant overall, the specific sentence that has a question or future action indication in *p_C_* may not be relevant to the suggestions in *p_B_*. In addition, the current technique for future action detection sometimes generates false positives. To address these issues, we will investigate how to leverage part-of-speech and reference resolution techniques [47] to improve natural language understanding.

### Conclusions and Future Work

We studied the problem of identifying influence relationships of web-based discussions and developed techniques and a prototype system for identifying influence relationships in OHCs. The proposed deep learning model demonstrates the performance advantage of the compared methods. As future work, we will address the aforementioned limitations to improve the generality and accuracy of the proposed techniques.

## Abbreviations

ARC-I: Architecture-I
ARC-II: Architecture-II
BERT: Bidirectional Encoder Representations from Transformers
CSN: Cancer Survivors Network
DSSM: Deep Structured Semantic Models
OHC: online health community
PR AUC: area under the precision-recall curve
ROC AUC: area under the receiver operating characteristic curve
